# Conceptualizing an inquiry-based lingua-cultural learning through telecollaborative exchanges

**DOI:** 10.12688/f1000research.55128.1

**Published:** 2021-07-28

**Authors:** Murod Ismailov

**Affiliations:** 1 Faculty of Humanities and Social Sciences, University of Tsukuba, Tsukuba, Ibaraki, 305-8577, Japan

**Keywords:** telecollaboration, inquiry-based learning, intercultural communication, intra-cultural learning, online exchanges, opinion paper

## Abstract

In recent years, telecollaboration has been gaining popularity among scholars, teachers, and students engaged in foreign language education because it facilitates the use of Internet-mediated communication tools to connect language and culture learners in geographically distant locations. Telecollaboration, as currently viewed in academic and classroom settings, places greater emphasis on the development of learners’ intercultural communicative competence. The problem with this approach is that this process may not consider the possibility that learners engaged in online intercultural exchanges could have limited or no knowledge about certain aspects of their own lingua-culture. We argue that, for learners to effectively share lingua-cultural knowledge with their online peers abroad, there must be a framework that supports the construction of learners’ own intra-cultural knowledge to provide a solid foundation for intercultural learning and communication. In this paper, we develop an inquiry-based model of telecollaboration incorporating both inquiry and online exchange based on the inquiry cycle, which includes engagement, exploration, explanation, elaboration, and evaluation. This paper builds a case for the application of inquiry-based telecollaboration in a real classroom environment, which could not only help learners obtain and eventually share more authentic, deeper knowledge about their lingua-culture, but also promote informed intercultural exchange.

## Introduction

In one of the early essays about the role of the Internet in foreign language instruction published in 1993, William J. Wyman pointed out that network technology would soon enable us ‘to do things differently, to learn in new ways, to see old things in new ways’ ( Wyman 1993, 28). This was the time when, in Wyman’s own words, a group of foreign language teachers invited to his workshop were serious when asking him questions, such as ‘How much will it cost me (or my department) to send email to Russia?’ or ‘By the way, can I send email to Russia?’ (26). More than 25 years later, foreign language teachers and their students can not only send emails to any person in any country in the world, but also engage in discussions; exchange ideas; make presentations; practice their speaking, writing, reading, and listening skills; and instantly get feedback from their partners through real-time communication applications ( Guth and Helm 2010).

In the academic literature, this form of interaction is known as telecollaboration, with some authors labelling it ‘an online intercultural exchange’ ( O’Dowd 2007) or ‘Internet-mediated intercultural foreign language education’ ( Belz and Thorne 2006). Telecollaboration is an important aspect of foreign language education because it highlights the importance of intercultural learning and communicative competence in the process of foreign language acquisition ( Byram 1997; O’Dowd 2012). Telecollaboration allows instructors to engage students through synchronous and asynchronous communication with representatives of other cultures in geographically distant locations by using free and accessible communication tools such as email, Skype, Zoom, or Google Hangouts. Such projects, ideally facilitated by their respective institutions, provide learners the opportunity to learn about other cultures and their socio-linguistic norms and patterns through interactions with peers while staying within the supportive context of their foreign language classroom ( O’Dowd 2012).

Thus, the main objective of such telecollaborative exchanges is not merely to provide a platform for language practice, but to promote the development of intercultural communicative competence among learners ( Byram 1997) through interaction, exchange ( Belz and Thorne 2006), and structured tasks ( O’Dowd and Waire 2009). In other words, the aim of online exchanges and, more broadly, foreign language instruction, is no longer to produce near-native speakers but, in the words of Byram (1997, 12), intercultural speakers who can ‘see and manage the relationships between themselves and their own cultural beliefs, behaviours, and meanings [...] and those of their interlocutors’. Therefore, it is suggested that telecollaboration pieces together language and inter- and intra-cultural learning to help learners become more effective communicators ( Belz and Thorne 2006).

However, our examination of existing telecollaboration cases suggests that the intra-cultural learning aspect of online exchange is the least academically researched. In other words, while the foreign language and intercultural learning aspects of telecollaboration have been extensively studied by scholars and practitioners, intra-cultural learning has not been sufficiently addressed by field experts. Telecollaboration places greater emphasis on the development of intercultural communicative competence but, in the majority of the works reviewed, rarely takes into account the possibility that learners engaged in online intercultural exchanges may not have sufficient awareness of their own lingua-culture. Latest research in telecollaboration reveals that many teachers and facilitators continue to ‘teach the same thing in a different way’ ( Kern, Ware, and Warschauer 2004), failing to help learners revisit the cultural precepts and phenomena within their own cultures.

In the telecollaboration context, we approach intra-cultural learning as a process of (re)discovering novel, sharable knowledge related to one’s own culture, language, and communicative patterns. It is an important constituent in evidence-based intercultural knowledge sharing between online exchange partners. The introduction of a functional framework for incorporating intra-cultural learning into telecollaborative projects could increase the effectiveness of online intercultural exchanges. We agree with Kern, Ware, and Warschauer (2004) that telecollaboration presents an opportunity for educators to use Internet-mediated tools not so much to teach the same thing in a different way, but to help learners carry out collaborative inquiry and acquire authentic knowledge by viewing their expanding identities and communication strategies as resources in the process.

In this study, we develop the notion of ‘inquiry-based telecollaboration’, as a practical framework for nurturing learners’ intercultural knowledge with the goal of developing their intercultural communication skills. Our study suggests that the practical application of inquiry-based telecollaboration could facilitate the development of foreign language learners’ essential skills of lingua-cultural inquiry and evidence-based knowledge sharing.

## Literature review

### Telecollaboration for foreign-language learning and intercultural communication

Information and communications technology bring people of different cultures and nations closer together, especially through common media such as the Internet. High-speed Internet communications provide foreign language educators with multiple instruction tools and resources that enable cost-effective, time-efficient, and productive lingua-cultural exchange and learning ( Kern, Ware, and Warschauer 2004; O’Dowd 2007; Guth and Helm 2010; Doolly 2017). Thorne (2010) points out that many of these new Internet-mediated learning practices that simultaneously incorporate multiple forms of media, such as text, voice, and video, used in both local and globally distributed settings, extend beyond traditional modes of information exchange dominated by print-based text.

Studies over the past two decades have provided important information on the benefits of telecollaboration, including expanding L2 pragmatic competence among foreign language learners ( Belz and Kinginger 2003), enhancing grammatical proficiency ( Lee 2002), vocabulary ( Dussias 2006), and spoken communication ( Abrams 2003). Other studies have reported positive outcomes in enhancing students’ independent learning (Schwienhost 2000) and developing multiliteracies that include digital, organisational, and critical skills ( Guth and Helm 2010).

The intercultural learning dimension of online exchanges is gaining prominence with culture and cultural knowledge playing a stronger role in the foreign language curriculum, and language learners being described as ‘cultural mediators’ and ‘intercultural speakers’ ( Byram 1997). Telecollaboration also highlights the importance of differentiating between multilingual and intercultural communication ( van der Kroon, Jauregi, and ten Thije 2015). Students learning a foreign language should not only develop grammatical competence, but also understand how language is used in a socially and culturally appropriate way. This awareness helps learners communicate and collaborate with speakers of other languages on equal terms, while simultaneously aware of their own identities and those of their exchange partners ( Savignon 2004).

Communication and collaboration are facilitated through telecollaborative tasks. O’Dowd and Waire (2009, 176-177) identified three main categories of tasks to systematise telecollaborative exchange: (1) information exchange, (2) comparison/analysis, and (3) collaboration/product creation. Information exchange tasks involve authoring ‘cultural autobiographies’, conducting virtual interviews, engaging in informal discussions, and exchanging stories. Comparison/analysis tasks include comparing parallel texts and class questionnaires, analysing cultural products, and translating. Collaboration/product creation includes working together to create products, transforming text genres, conducting ‘closed outcome’ discussions, and making cultural translations/adaptations.

### Telecollaboration and intra-cultural learning

The introduction of the concept of intercultural communicative competence has led to a focus on raising learners’ awareness of how their own cultural background and assumptions may impact their attitudes towards and communication with people from other cultures ( Alred, Byram, and Fleming 2003; Corbett 2003). A key characteristic of intercultural communicative competence is the fact that it prepares learners for exposure to all cultures, including their own ( Mughan 1999, 64). This is where the notion of intra-cultural communication gains prominence; thus, at least in theory, it should emerge as a crucial component in online intercultural exchange projects.

Samovar and Porter (2001) defined intra-cultural communication as communication that takes place between members of the same dominant culture, but with different values, as opposed to intercultural communication, which is the communication between two or more distinct cultures. According to Kecskes (2012, 68), this approach has led to a common but misinformed perception of inter-culturality as the main reason for miscommunication ( House 2003; Kecskes 2008). Wray (2002) suggested that intra-cultural communication is dominated by preferred ways of saying things and, according to Kecskes (2008), of organising thoughts within a particular speech community. This occurs differently in intercultural communication because the development of ‘preferred ways’ requires time and conventionalisation within a speech community due to the flexibility of human languages ( Kecskes 2012).

Intra-cultural communication is intertwined with the notion of intra-cultural learning. All knowledge and information is formed or received by learners at a certain time via family, school, media, or broader societal exposure ( Pinker 1999). This is especially true regarding learners’ own culture, native language, and norms of communication. This knowledge forms unwittingly through daily repetitive exposure, as evidenced by an average, healthy child less than six years who already can speak their native language without ever attending a language class ( Chaney 2001). Typically, by early adolescence, this native lingua-cultural knowledge has already been formed to the extent that it allows the person to communicate with other members of the same community not only grammatically, but also in culturally appropriate ways. This process of native lingua-cultural knowledge development slows by early adulthood, but does not end completely ( Crawford-Lange and Lange 1987).

Learners acquire knowledge of their own language and culture dynamically, and the search for new knowledge stems from their motivation, expertise, and future career choices, especially among professional linguists, public speakers, and culturologists ( Breen 1985). This is also true with regard to telecollaboration and online intercultural exchanges from which participants gain original insights of not only their peers’ language – manifested in the way they speak, pronounce words, and form expressions – but also their culture – the way they live, do things, articulate ideas, and understand concepts ( Lamy and Goodfellow 2010). Many participants of online lingua-cultural exchanges share information about their language and culture based on their pre-existing, often limited, lingua-cultural knowledge.

Paradoxically, the abundance of highly specialised but easily searchable knowledge on the Internet ( Jones and Kucker 2001) for anyone possessing a smartphone or computer may be the reason for Internet-mediated language exchanges eventually turning into a demotivating activity in which participants’ expectations of new cultural and linguistic knowledge are not met during telecollaborative interactions. Thus, online language and intercultural exchanges should be designed considering learners’ awareness of their own culture and language, its uniqueness and originality and, importantly, the level of knowledge credibility to be shared between online exchange partners.

Byram (1997, 53) proposed the concept of ‘critical cultural awareness’, defined as an ability to evaluate critically and on the basis of explicit criteria, perspective, practices and products in one’s own and other cultures and countries. Similarly, Risager (2007) suggested that intercultural competence comprises knowledge, skills, and attitudes at the interface between several cultural areas, including the students’ own culture and a target culture.

Our review of the literature showed that telecollaboration to date has been extensively studied in relation to intercultural communication, but no major studies have been conducted to explore a systematic connection between online exchange and intra-cultural communication and learning. In the next section, we introduce the inquiry-based model of telecollaboration aimed at filling this gap, thus yielding both conceptual and practical implications for designing online intercultural exchanges.

## Inquiry-based model of telecollaboration

**Figure 1.**
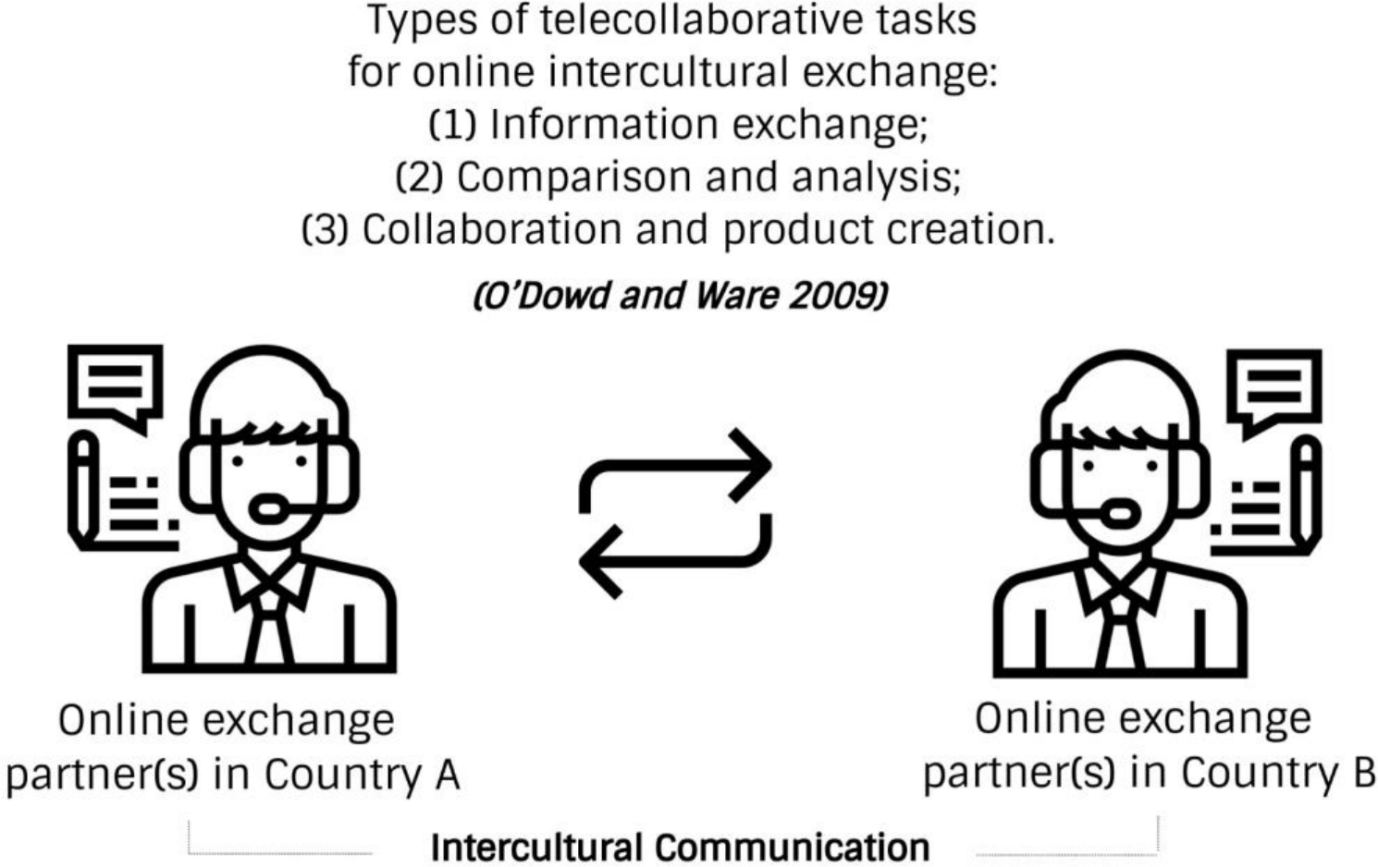
Conventional online exchange and telecollaborative tasks.

Existing telecollaboration cases reveal that online intercultural exchange necessitates the presence of two or more linguistically and culturally distinct groups of individuals whose goal is to learn about each other’s language and culture through various Internet-mediated activities, such as information exchange, comparison and analysis, and collaboration and product creation (see Figure 1).

In this conventional form, learners on both sides consider themselves representatives of their culture and native speakers of their language, viewing each other as possessors of authentic cultural and linguistic knowledge. We encounter four problems related to this approach. First, the cultural knowledge that a host member shares with a foreign member is often subjective, especially related to activities involving interpretation, contextualisation, comparison, and negotiation of meaning. However, online exchange may be less susceptible to subjectivity when partners engage in a rather technical language exchange, such as vocabulary learning, sentence construction, and practising correct pronunciation and grammar.

The second problem arises when the limited online interactions further augment learners’ stereotypical view of their partners’ culture. This impacts learners on both sides: foreign members solidify their stereotypes based on limited information gained through a short exercise, while host members remain convinced that these stereotypes concerning certain aspects of their lingua-culture are well-grounded because of insufficient intra-cultural knowledge. The third problem is related to foreign member generalising issues based on their interaction with a limited number of ‘host’ members.

The fourth and perhaps more salient problem is that the majority of telecollaborative tasks reported to date tend to initiate intercultural contacts without addressing the host members’ lack of knowledge about their own lingua-culture. This creates a learning environment in which foreign members may find it difficult to acquire authentic knowledge about the target culture, and host members might miss the opportunity to expand their knowledge reservoir of their own culture, which lies at the core of building intra-cultural competence. An integral part of becoming an intercultural speaker is intra-cultural learning; that is, learning about one’s own culture and developing the ability to reflect on the origin of one’s own beliefs and behaviours.

These combined problems present a challenge in the effective use of Internet-mediated tools for developing intercultural communicative competence. The modern multimedia-rich Internet offers enough terabytes of knowledge on any of the world’s cultures and languages to render the whole idea of online intercultural exchanges more burdensome to some learners, unless there is a framework that could help online exchanges pro-actively develop intercultural communicative competence. The question that we pose here, therefore, is: how could telecollaborative activity be re-designed to minimise the impact of these factors on the development of intercultural communicative competence aimed at online exchanges? Addressing this question brings us to intra-cultural communication and learning, or to be exact, their paucity in the design of most contemporary telecollaborative projects.

One important yet insufficiently explored area concerns the relationship between inquiry and the creation of a new lingua-cultural knowledge. In our proposed model, we closely examine inquiry-based learning because it represents a more systemic and scientific way of constructing new knowledge by following certain procedures, methods, and practices ( Keselman 2003). Inquiry-based learning according to Pedaste *et al*. (2012) is a process of discovering new causal relations, with the learner formulating hypotheses and testing them by conducting experiments and/or making observations. Several studies support the effectiveness of inquiry-based learning as an instructional approach, suggesting that it can improve different skills, such as identifying problems, formulating questions, collecting and analysing authentic information, and presenting results and conclusions ( Alfieri *et al.* 2011; Furtak *et al.* 2012).

**Figure 2.**
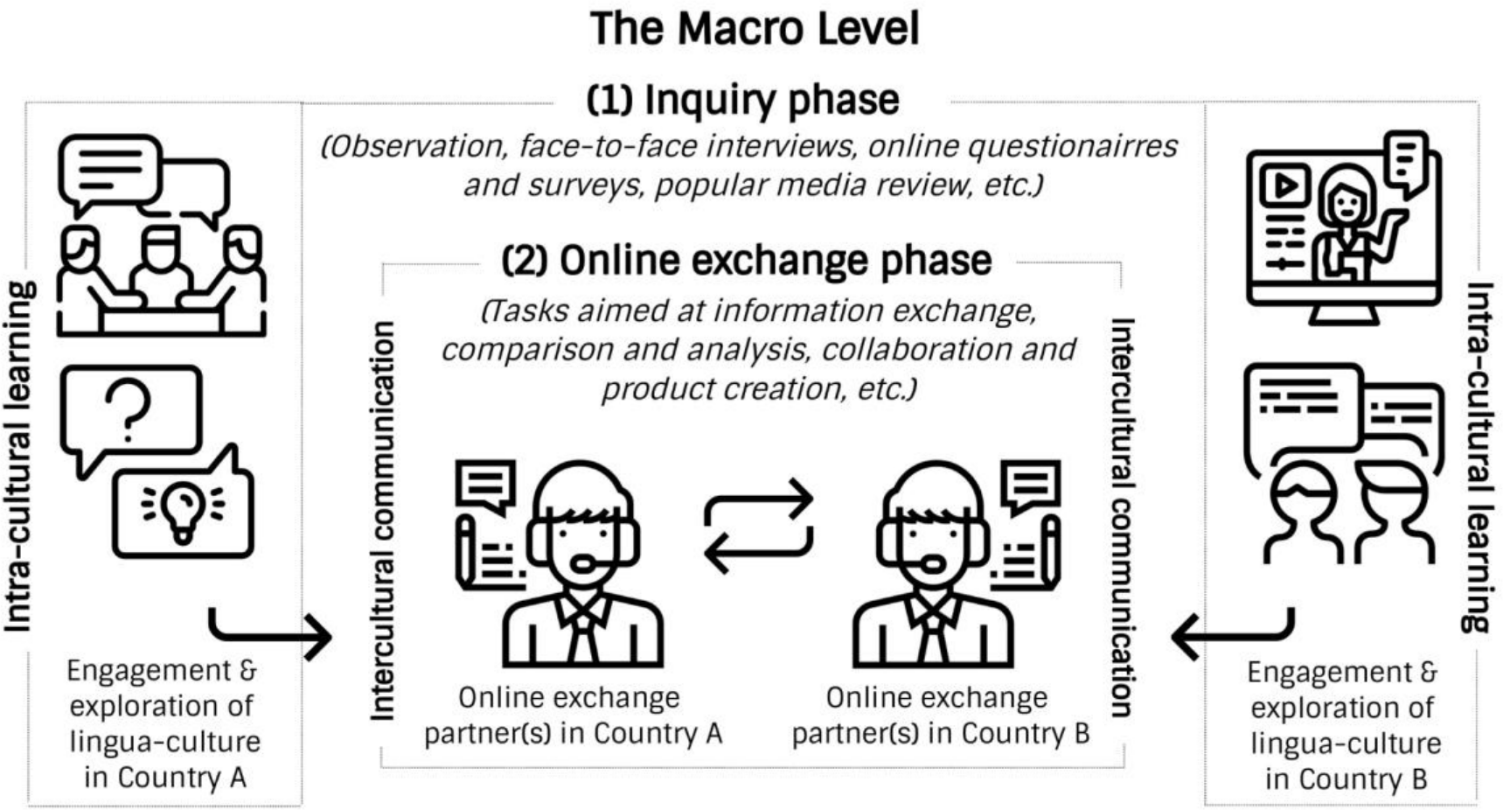
The inquiry-based model of telecollaboration (macro level).

Because our main concern about the effective implementation of telecollaboration as a medium of intercultural learning is the possible lack of host members’ knowledge of their own culture, the inquiry methods, especially those related to observation, identifying problems, formulating questions, and conducting experiments (interviews, surveys, etc.) provides a framework for the construction of intra-cultural knowledge aimed at elevating learners’ self-reflection and self-awareness. This telecollaboration model thus introduces an important new phase in the online exchange process that is designed to help host members discover authentic intra-cultural knowledge through inquiry within their own lingua-cultural environment (see Figure 2).

An inquiry-based model of online intercultural exchange can promote active learning because participants are expected to collect authentic knowledge by engaging members of their own community and then share the findings with their online exchange partners. Inquiry-based activity creates authentic contexts, and research in cognitive science highlights its role in effective learning ( Greeno, Collins, and Resnick 1996). Edelson, Gordin, and Pea (1999) claim that authentic activities provide learners with the motivation to acquire new knowledge, an opportunity to incorporate it into their existing knowledge, and conditions in which to apply this knowledge in real life. While the transmission and reception of knowledge in conventional online intercultural exchange is passive, an inquiry-based model is active. As a practise aimed at discovering authentic perspectives within one’s own culture (stereotypes, perceptions, beliefs, norms of behaviour) and language (patterns of verbal and non-verbal communication, geographic peculiarities of language), inquiry-based telecollaboration also promotes informed intercultural communication.

It is important to mention that in our proposed telecollaboration model, the ‘inquiry phase’ and the ‘online exchange phase’ are not mutually independent processes; they are organically intertwined to provide a cohesive learning experience for online exchange partners. To understand this connection, one should recognise that inquiry itself is a process containing smaller, logically connected units (phases) that guide learners and draw attention to important features of scientific thinking. This set of connections represents an inquiry cycle ( Pedaste *et al.* 2015).

Various academic studies of inquiry-based approaches to learning and teaching have attempted to propose different versions of an inquiry cycle. For example, in their meta-analysis of the strengths of over 30 inquiry-based learning frameworks, Pedaste *et al.* (2015) suggested five distinct general inquiry phases: orientation, conceptualisation, investigation, conclusion, and discussion. An inquiry cycle developed by White and Frederiksen (1998) also identified five inquiry phases, labelled question, predict, experiment, model, and apply.

Despite the numerous variations in learning cycles, the one used in this study is the 5E Instructional Model ( Bybee *et al.* 2006; Bybee 2009) in which each phase is highlighted using five words: engagement, exploration, explanation, elaboration, and evaluation. This approach is useful for inquiry-based telecollaboration design because it provides a format for online exchange that builds on what learners already know. Since the average host members already possess some knowledge of their lingua-culture, the 5E Instructional Model helps them revisit that knowledge from other angles and find new patterns and relationships. Moreover, the experience of undergoing all five phases enables online exchange participants to develop their understanding of a concept across time.

The 5E Instructional Model emphasises not only students’ hands-on knowledge acquisition and learning, but also the role of instructors in facilitating this process, which is why it is known as an instructional model. The role of teachers is reflected in a widely cited definition of telecollaboration as ‘institutionalised, electronically mediated intercultural communication under the guidance of a lingua-cultural expert (i.e. teacher) for the purposes of foreign language learning and the development of intercultural competence’ ( Belz 2003).

Even in the context of conventional telecollaborative projects in which a teacher's active involvement in conducting online tasks is not necessary because learners typically interact entirely with their distant partners ( O’Dowd 2013), there is considerable need for instructors’ indirect participation and regular guidance. Right from the early stages of the project, teachers are responsible for designing tasks, choosing tools, and establishing the rules of engagement and a timeframe for self-reflection and group feedback in order to enable productive collaboration and communication between two or more socio-linguistically and culturally distinct groups of learners ( Lewis, Chanier, and Youngs 2011). Because inquiry-based telecollaboration adds an additional layer to the design of an online exchange project, that is ‘intra-cultural inquiry’ phase, teachers’ guidance becomes even more significant.

**Figure 3.**
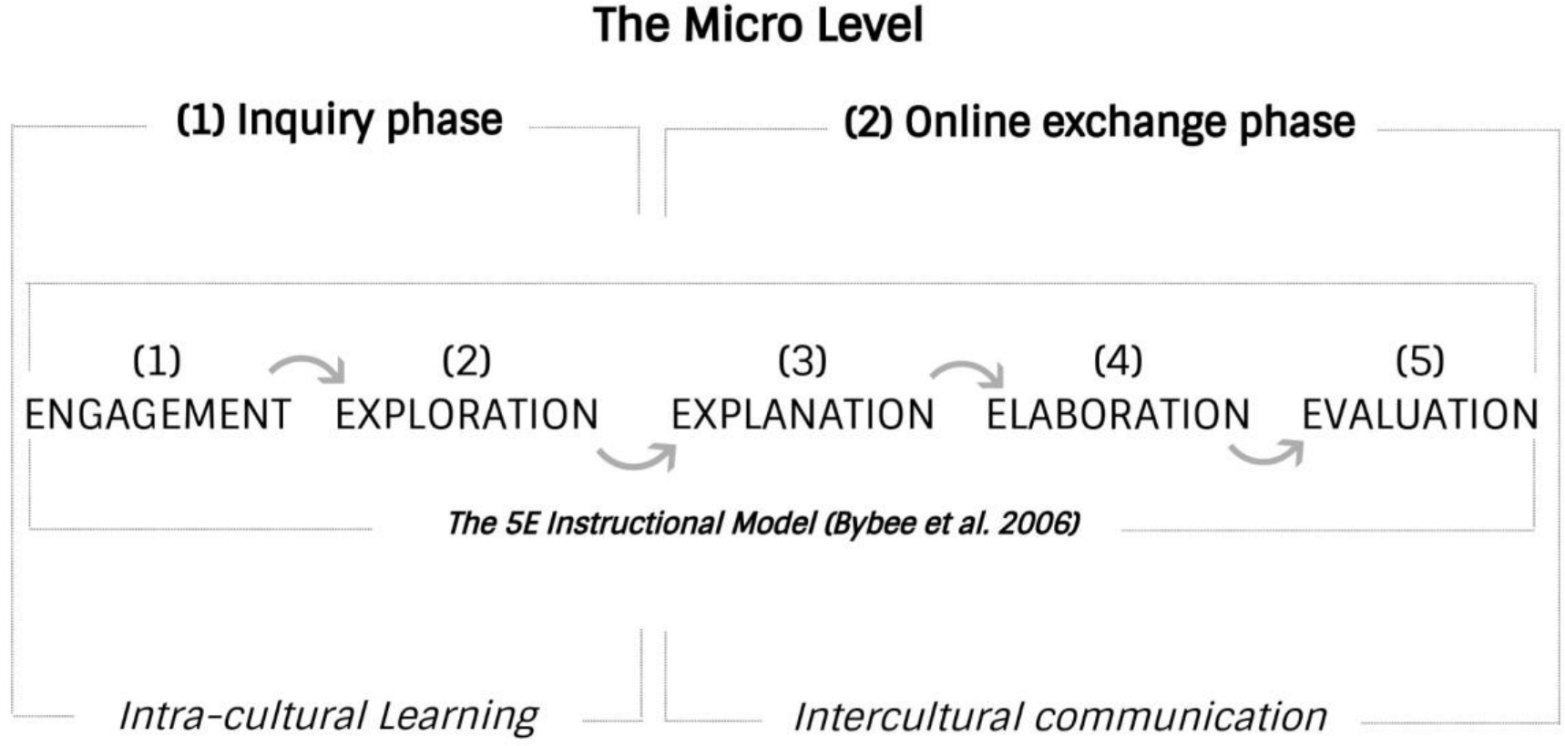
The inquiry-based model of telecollaboration (micro level).

The inquiry cycle contains multiple units that can be rolled out across both ‘inquiry’ and ‘online exchange’ phases (as shown in Figure 3), thereby creating a holistic framework that connects intra-cultural learning with the process of developing intercultural communicative competence. Below, we describe the five phases of inquiry-based telecollaboration that have been modified from the 5E Instructional Model. To illustrate the main points of our approach, we shall also use the hypothetical example of a telecollaborative project conducted between two groups of college students based in Japan and the US on the socio-cultural topic of ‘Parenting culture in Japan and the US: differences and similarities’. We assume that both groups have upper-intermediate knowledge of their exchange partners’ language, that is, Japanese and English, respectively. During this hypothetical project, participants virtually interviewed their partners, with the aim of practicing linguistic skills and acquiring deeper knowledge about their culture.

### The engagement phase

In this initial ‘inquiry’ phase, teachers work closely with their students to evaluate their prior knowledge and identify possible gaps in their current understanding of the topic. The key is that students at this phase are focussed on the knowledge and knowledge gaps relating to their own culture and society, not those of their online partners. Through visual demonstrations, questioning sessions, and graphic organisers, teachers help students clarify their thinking, make connections to prior knowledge and what is to be discovered, and stay mentally engaged in the new learning experience. One of the key responsibilities of the inquiry-based telecollaborative teacher is to stimulate interest and generate curiosity in their learners throughout the project. Thus, in our hypothetical telecollaborative project, the Japanese students may start brainstorming with an opening question ‘What do we already know about parenting in Japan?’. American students, on the other hand, ask ‘What do we already know about parenting in the US?’. One effective technique may involve the use of real KWL charts, wherein learners make a list of what they already Know, Want to know, and (eventually) have Learnt about the topic.

### The exploration phase

The students now proceed to the ‘core’ inquiry phase, involving an active exploration of the issue in the context of their own culture. In other words, they begin building essential intra-cultural knowledge by searching for authentic information. In terms of knowledge seeking, learners continue to stay within the boundaries of their own lingua-culture. This increased awareness about the specific aspect (topic) of their culture eventually helps both them and their online exchange partners to develop more effective intercultural understanding and communication. In this phase, students are encouraged to learn and apply core inquiry skills, such as observing, questioning, investigating, testing predictions, hypothesising, and eventually communicating with their online exchange partners.

In the context of our hypothetical telecollaborative project, both Japanese and American students (individually or in teams) will engage in real-life observations and collect and record data through face-to-face or online interviews, Facebook or Twitter polls, focus group interviews, and so forth. For example, one group of Japanese students may decide to conduct face-to-face interviews with their parents and grandparents in order to better understand the peculiarities of parenting culture in Japan, whereas another group may organise an opinion poll of younger and older generations of parents using a popular instant messaging application, LINE. In both cases, students will have to design structured, semi-structured, or open-ended questionnaires based on the work conducted during the ‘engagement phase’ (e.g. KWL charts).

The exploration phase of the project possibly incorporates the most critical inquiry-based experience aimed at developing students’ essential intra-cultural knowledge. This phase is unique because the students get a ‘hands-on’ experience collecting authentic intra-cultural insights based on real-world observations and explorations. Although they are encouraged to work in a co-operative learning environment without direct instruction from the teacher, teachers nonetheless actively guide students in their inquiry, regularly asking probing questions to enhance their students’ understanding.

### The explanation phase

In inquiry-based telecollaboration, the explanation phase signals the beginning of the ‘online exchange phase’, in which learners from project countries (cultures) connect with the aim of explaining the results of their intra-cultural inquiry. Students work together in pairs or small groups and verbalise their understanding of the information gathered in their respective countries during the exploration phase, seek and analyse patterns in their data, and describe what they observed. Teachers, on the other hand, begin playing more subtle roles, allowing students to actively interact with each other.

During this phase, the Japanese and American participants of our hypothetical project connect with each other using Skype. The teachers previously created small groups and assigned two American and two Japanese students to each of the groups. The goal of this interaction is to conduct virtual interviews during which American students have the opportunity to share their observations and knowledge pertaining to parenting culture in their home country with their Japanese partners, and vice versa. The difference between conventional and inquiry-based telecollaborative environments is that the latter provides a stronger intra-cultural knowledge base, allowing for informed intercultural dialogue between the students. New insights and ideas are likely to be generated by the time participants complete the explanation phase.

### The elaboration phase

The activities in this second ‘online exchange’ phase are designed to help exchange partners continue to collaborate in order to apply their new understanding of cultural concepts (similarities and differences in parenting cultures between two countries) shared by each country member. Students are encouraged to compare notes with their peers or formulate new observations of the concepts they have acquired. The goal of this phase is to help learners develop a deeper and broader understanding of each other’s cultural concepts. With this in mind, our hypothetical virtual groups of Japanese and American students conduct additional collaborative investigations (e.g., social media polls, surveys, mass media analyses, etc.) or activities such as reading articles and books, writing a blog post, creating web pages, or exploring related topics on the Internet through WebQuests. Teachers’ engagement remains minimal.

### The evaluation phase

The evaluation phase may take different forms, it may continue as an ‘online exchange’ activity or students may return to their respective classrooms to assess the project’s achieved objectives. The aim is to encourage students to reflect on what they have learnt about their own and their partners' culture, pose questions, and illustrate their knowledge (understanding) and skills (abilities). For example, after completing online presentations, both American and Japanese students may return to their KWL charts and complete the ‘What I Have Learnt’ part. In this phase, teachers play an important role in evaluating the project’s success. The use of various forms of assessment – such as portfolios, concept maps, group presentations, and journal logs – may serve as important evidence of student learning. Students can also conduct self- or peer-assessments. This phase may also incorporate a summative assessment such as a quiz, test, or writing assignment. Teachers may conduct class surveys with the goal of understanding the problems students face during both intra-cultural learning and intercultural online exchange.

These five phases of inquiry-based telecollaboration represent a holistic framework that can practically help foreign language learners gain in-depth knowledge of their own lingua-culture in order to support more engaging intercultural communication with peers on a global level, and develop essential skills of inquiry and evidence-based knowledge sharing. The future development of this model could demystify the image of ‘inquiry-based learning’ as a method used mainly in science labs, instead promoting it as a practical and effective learning method in social sciences and humanities.

## Conclusion

Since its early emergence in the 1990s, telecollaboration has been gaining popularity among foreign language and culture learners mainly because of its broad use of communication technologies and the Internet to connect with lingua-cultures globally. With technological advancements, which began placing greater emphasis on user-generated content, ease of use, participatory culture, and interoperability for end users, as reflected in the phenomenon known as Web 2.0, it was inevitable that telecollaboration would soon follow suit. In 2010, a group of renowned experts joined forces and published a volume titled ‘Telecollaboration 2.0: Language, Literacies and Intercultural Learning in the 21st Century’ ( Guth and Helm 2010), reflecting the prevailing role of communication technologies and the Internet in the design and effective execution of telecollaborative projects.

On the contrary, the broader aim of inquiry-based telecollaboration is to deemphasise the role of technology and the Internet in intercultural education, intending instead to accentuate the importance of acquiring authentic lingua-cultural knowledge and, in Byram’s words, develop ‘critical cultural awareness’ (1997, 35). Technology is only a medium, albeit an important one, whose underlying goal is to help learners access such lingua-cultural knowledge in a fast, engaging, and cost-effective way.

Despite its relatively straightforward design, several questions still remain regarding the real-life implementation of the proposed model of telecollaboration. Therefore, future research on inquiry-based telecollaboration should address the issues that can be placed on four different levels, in accordance with the inventory of possible reasons for the breakdown of conventional telecollaborative exchanges ( O’Dowd and Ritter 2006): the individual level (learners’ attitudes and motivation during ‘inquiry’ and ‘online exchange’ phases), the classroom level (how inquiries and exchanges were organised and implemented in both classes, and which telecollaborative tasks proved effective when incorporated within the inquiry model), the socio-institutional level (different levels of access to technology, challenges in designing inquiry and experiments, institutional attitudes toward online learning), and the interaction level (the quality and outcomes of intercultural communication that occurred between exchange partners). Answers to these questions may be found through continuous comprehensive studies that could potentially shed light on other crucial aspects of Internet-mediated intercultural exchanges.

## Data availability

No data is associated with this study.
