# Supplementary material for: Conceptualizing an inquiry-based lingua-cultural learning through telecollaborative exchanges
Source: F1000Res. 2021 Sep 2;10:677. Originally published 2021 Jul 28. [Version 2] doi: 10.12688/f1000research.55128.2 (PMC8371411; doi:10.12688/f1000research.55128.2)
Supplement: Peer review history

# Peer review: Reviewer response for version 1

**DOI:** 10.5256/f1000research.58674.r91517

**Published:** 2021-08-25

**Authors:** Pramila Neupane

**Affiliations:** 1 University Education Center, Gunma University, Maebashi, Japan

The author examines the telecollaborative exchanges in foreign language education and emphasizes the importance of intra-/inter-cultural language learning for building intercultural communicative competence. Using the 5E Instructional Model suggested by Bybee et al. (2006), the author conceptualized an inquiry-based model of telecollaboration. This paper is a timely contribution by the author as telecollaboration is gaining popularity in teaching and learning in various fields in recent years. 

 The author's opinion and arguments would be stronger if the author could discuss how the model works in different teaching/ learning environments. I recommend this manuscript for indexing.

Is the topic of the opinion article discussed accurately in the context of the current literature?

Yes

Are arguments sufficiently supported by evidence from the published literature?

Yes

Are all factual statements correct and adequately supported by citations?

Yes

Are the conclusions drawn balanced and justified on the basis of the presented arguments?

Yes

Reviewer Expertise:

Comparative and International Education; Education Policy; Language Education

I confirm that I have read this submission and believe that I have an appropriate level of expertise to confirm that it is of an acceptable scientific standard.

# Peer review: Reviewer response for version 1

**DOI:** 10.5256/f1000research.58674.r90631

**Published:** 2021-08-17

**Authors:** Sherzod Muminov

**Affiliations:** 1 University of East Anglia, Norwich, UK

This is an innovative paper that draws attention to a gap in scholarship on the role of intra-cultural knowledge in building effective telecollaborations between learners from different cultures. The author rightly underlines the importance of intra-cultural knowledge and understanding, and not simply of intercultural differences, in establishing mutually beneficial telecollaborations in various educational settings. The paper then proposes a new inquiry-based model of intercultural communication. The model is well defined and explained through a five-phase process, with real-life examples and a lucid analysis of impacts and outcomes. As such, this paper contributes to current knowledge on telecollaborations and potentially in other fields, and should be recommended for indexing.

Is the topic of the opinion article discussed accurately in the context of the current literature?

Yes

Are arguments sufficiently supported by evidence from the published literature?

Yes

Are all factual statements correct and adequately supported by citations?

Yes

Are the conclusions drawn balanced and justified on the basis of the presented arguments?

Yes

Reviewer Expertise:

Humanities, specifically history

I confirm that I have read this submission and believe that I have an appropriate level of expertise to confirm that it is of an acceptable scientific standard.

# Peer review: Reviewer response for version 1

**DOI:** 10.5256/f1000research.58674.r90630

**Published:** 2021-08-02

**Authors:** Rustamjon Urinboyev

**Affiliations:** 1 Department of Sociology of Law, Lund University, Lund, Sweden

The author explores how intra-cultural language learning occurs through telecollaborative exchanges. He has rightly identified the gap in the literature, which is an understudied topic of the systematic connection between the online exchange and intra-cultural communication and learning. To fill this gap, the author proposes an inquiry-based model of telecollaboration, incorporating both inquiry and online exchange through macro- and micro- levels. One small comment would be to emphasize the argument in the section 'Conclusion'. I recommend this manuscript for indexing.

Is the topic of the opinion article discussed accurately in the context of the current literature?

Yes

Are arguments sufficiently supported by evidence from the published literature?

Yes

Are all factual statements correct and adequately supported by citations?

Yes

Are the conclusions drawn balanced and justified on the basis of the presented arguments?

Yes

Reviewer Expertise:

Social sciences

I confirm that I have read this submission and believe that I have an appropriate level of expertise to confirm that it is of an acceptable scientific standard.
